# Dopamine and vesicular monoamine transport loss supports incidental Lewy body disease as preclinical idiopathic Parkinson

**DOI:** 10.1038/s41531-023-00514-z

**Published:** 2023-06-15

**Authors:** Christian Pifl, Harald Reither, Johannes Attems, Luigi Zecca

**Affiliations:** 1 Center for Brain Research, Medical University of Vienna, Vienna, Austria; 2 Translational and Clinical Research Institute, Newcastle University, Newcastle upon Tyne, UK; 3 Institute of Biomedical Technologies, National Research Council of Italy, Segrate, Milan, Italy

**Keywords:** Parkinson's disease, Transporters in the nervous system

## Abstract

Incidental Lewy body disease (ILBD) is a neuropathological diagnosis of brains with Lewy bodies without clinical neuropsychiatric symptoms. Dopaminergic deficits suggest a relationship to preclinical Parkinson’s disease (PD). We now report a subregional pattern of striatal dopamine loss in ILBD cases, with dopamine found significantly decreased in the putamen (−52%) and only to a lower extent in the caudate (−38%, not statistically significant); this is similar to the pattern in idiopathic PD in various neurochemical and in vivo imaging studies. We aimed to find out if our recently reported impaired storage of dopamine in striatal synaptic vesicles prepared from striatal tissue of cases with idiopathic PD might be an early or even causative event. We undertook parallel measurements of [^3^H]dopamine uptake and vesicular monoamine transporter (VMAT)2 binding sites by the specific label [^3^H]dihydrotetrabenazine on vesicular preparation from caudate and putamen in ILBD. Neither specific uptake of dopamine and binding of [^3^H]dihydrotetrabenazine, nor mean values of the calculated ratios of dopamine uptake and VMAT2 binding, a measure of uptake rate per transport site, were significantly different between ILBD and controls. ATP-dependence of [^3^H]dopamine uptake revealed significantly higher rates in putamen than in caudate at saturating concentrations of ATP in controls, a subregional difference lost in ILBD. Our findings support a loss of the normally higher VMAT2 activity in putamen as a contributing factor to the higher susceptibility of the putamen to dopamine depletion in idiopathic PD. Moreover, we suggest ILBD postmortem tissue as a valuable source for testing hypotheses on processes in idiopathic PD.

## Introduction

Individuals with Lewy bodies or Lewy neurites at autopsy but without clinical findings of parkinsonism or dementia during their lifetime can be subsumed under cases with incidental Lewy body disease (ILBD). They are considered pre-symptomatic Parkinson’s disease (PD) by their nigrostriatal pathological features that are intermediate between those in morphologically normal control persons and those with overt PD^1,2^. However, there is a range of neurological syndromes with striatal loss of dopamine that are distinct from idiopathic PD, such as multiple system atrophy and progressive supranuclear palsy^3–6^, summarized as atypical PD. In neurochemical studies on postmortem brains of patients with idiopathic PD, a characteristic pattern of dopamine loss has been found in the striatum with a nearly complete depletion of dopamine in all subdivisions of the putamen and substantial levels of dopamine remaining in the caudate^7^. With the progress of in vivo imaging methods, this pattern has also been detected in live patients with various stages of idiopathic PD^8–12^ and allowed differentiation between idiopathic PD and atypical PD^13–17^. Although the incidence of idiopathic PD increases with age, and there is an age-dependent reduction in striatal dopamine innervation^18^, the regional pattern of striatal dopamine loss in normal aging differs substantially from the pattern typically observed in idiopathic PD^19^. By contrast, 18F-dopa uptake for asymptomatic co-twins of patients with PD already revealed the typical pattern with a small but significant reduction of the dopamine marker in putamen but not in caudate^20^.

In ILBD, two neurochemical markers of striatal dopamine nerve terminals have been reported to be reduced, the immunoreactivity of tyrosine hydroxylase^1,2,21^ and the vesicular monoamine transporter 2^1^. However, all striatal findings are based on analysis of the putamen, and there are neither data on the caudate in ILBD in the literature nor on striatal dopamine tissue levels. We therefore set out for analysis of monoamine neurotransmitter tissue levels in both caudate and putamen of cases with ILBD. In a recent study on postmortem striatal tissue of cases with idiopathic PD, we observed a loss of 56% and 90% dopamine transport efficiency in synaptic vesicles of dopamine nerve terminals in the caudate and in the putamen, respectively^22^. Therefore, we also performed vesicular uptake on the caudate and putamen of our cases in this presumptive preclinical phase of PD.

## Results

### Monoamine neurotransmitters

**Fig. 1:**
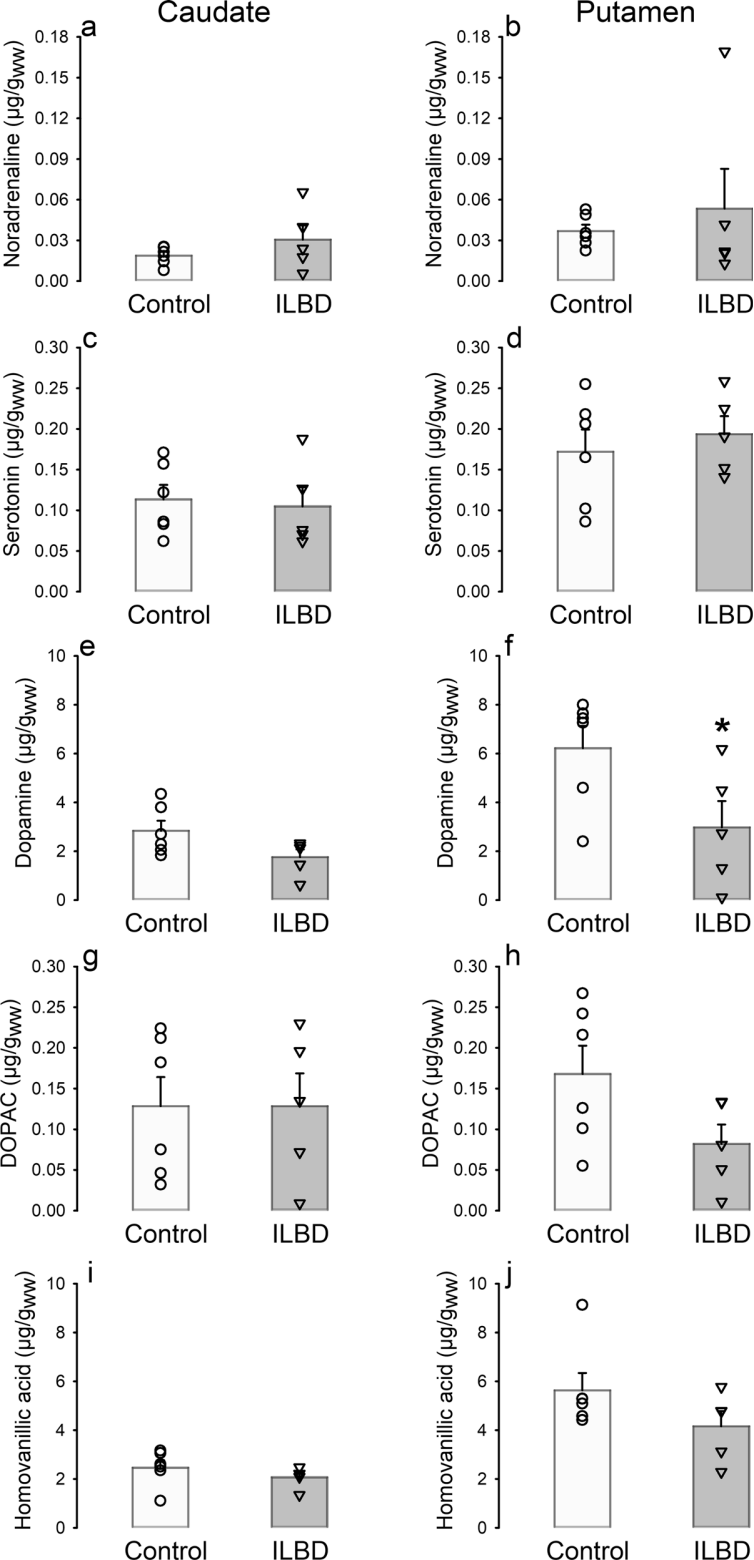
Monoamine neurotransmitters and dopamine metabolites. Tissue levels of noradrenaline (**a**, **b**), serotonin (**c**, **d**), dopamine (**e**, **f**), and its metabolites 3,4-dihydroxyphenylacetic acid (**g**, **h**) and homovanillic acid (**i**, **j**) in caudate (left panels) and putamen (right panels) of control and ILBD subjects. Data are given as single values, means and the error bar s.e.m. in µg/g wet weight. *p < 0.05 vs. control by two-way analysis of variance for the factors disease (ILBD or control) and region (caudate or putamen).

Monoamine neurotransmitter levels in striatal tissue of control cases (Fig. 1a–f) displayed the typical concentration range with mean values of serotonin about five times and of dopamine about 150 times higher than that of noradrenaline^23–25^. Whereas noradrenaline and serotonin were either unchanged or insignificantly increased in ILBD (Fig. 1a–d), two-way analysis of variance for the factors disease (ILBD or control) and region (caudate or putamen) revealed a significant decrease of dopamine in the putamen (−52%, *t* = 3.044, *p* = 0.007; Fig. 1f), but not in the caudate (−38%) in ILBD (Fig. 1e).

**Fig. 2:**
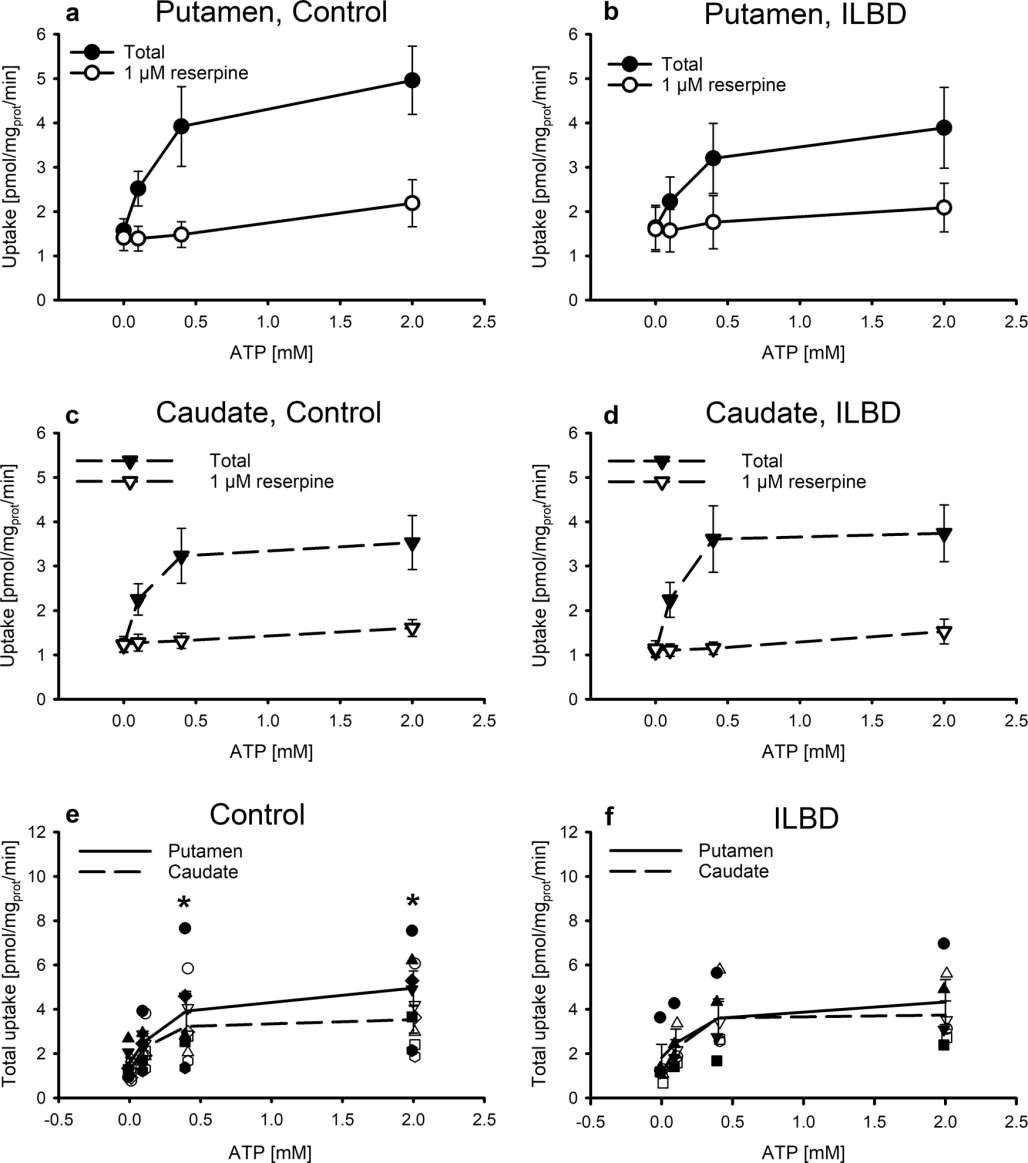
ATP-dependence of vesicular uptake. Dopamine uptake by synaptic vesicles prepared from striatal tissue of control (left panels) and ILBD subjects (right panels). Total uptake (closed symbols) and uptake in the presence of reserpine (open symbols) in the putamen (**a**, **b**) and in the caudate (**c**, **d**) and comparison of total uptake in control (**e**) and ILBD subjects (**f**) of the putamen (closed symbols) vs. caudate (open symbols). Synaptic vesicle preparations were incubated with 0.1 µM [^3^H]dopamine and MgATP at the concentrations indicated at 30 °C for 5 min in the absence or presence of 1 µM reserpine. Data are given as means ± s.e.m. (controls, *n* = 6; ILBD, *n* = 4) and additionally single values in (**e**, **f**). *p < 0.05 vs. caudate by two-way repeated measures ANOVA for the factors region (caudate or putamen) and ATP (0, 0.1, 0.4, or 2 mM).

### Dopamine metabolites

The dopamine metabolites DOPAC and HVA have not been found to change significantly in ILBD, with mean values of ILBD 0.1% and 16% below controls, respectively, in caudate (Fig. 1g, i) and 51% and 26% below controls, respectively, in the putamen (Fig. 1h, j). Molar ratios of HVA/dopamine were quite similar in the caudate and putamen of control cases (caudate, 0.77 ± 0.11; putamen, 0.89 ± 0.20), whereas the respective mean value was strongly but insignificantly increased in the putamen of ILBD with high variation between single cases (caudate, 1.13 ± 0.19; putamen, 4.69 ± 3.28).

### Dopamine uptake and DTBZ binding in striatal synaptic vesicles

In order to determine transport efficiency in striatal synaptic vesicles, we isolated vesicular preparations of caudate or putamen tissue following our recent protocol^22^ and performed parallel dopamine uptake experiments and binding experiments with [^3^H]DTBZ, a label selective for the vesicular monoamine transporter 2 (VMAT2). The calculated ratio of dopamine uptake to [^3^H]DTBZ binding reflects the number of transported dopamine molecules per unit time per VMAT2 transport site, a measure of turnover or transport efficiency. There was no significant difference between the putaminal or caudate tissue of controls and ILBD cases neither in dopamine uptake and [^3^H]DTBZ binding nor in turnover (Table 1).

**Table 1:** Dopamine uptake and vesicular monoamine transporter binding sites in synaptic vesicular preparations from caudate and putamen of control subjects and cases with ILBD.

	Caudate	Putamen
[^3^H]dopamine uptake		
Control	3.32 ± 1.45	2.69 ± 1.17
ILBD	3.73 ± 0.82	3.10 ± 0.89
[^3^H]DTBZ binding		
Control	0.99 ± 0.23	1.06 ± 0.34
ILBD	1.12 ± 0.07	0.88 ± 0.22
Uptake/binding site		
Control	2.93 ± 0.57	2.39 ± 0.35
ILBD	3.48 ± 0.60	3.39 ± 0.40

Data ([^3^H]dopamine uptake, pmol/mg_prot_/min; [^3^H]DTBZ binding, pmol/mg_prot_; uptake/binding site, min^−^^1^) represent mean ± SE values (*n* = 5).

### ATP-dependence of vesicular dopamine transport

There was a considerable variation between single cases of control and ILBD samples, as is often the case in biological assays on postmortem tissue. Therefore, we went for parallel measurements of ATP-dependency of dopamine uptake in the putamen and caudate tissue for each control and each ILBD case at 0, 0.1, 0.4, and 2 mM ATP using a shortened preparation of synaptic vesicles without ATP preincubation. In the absence of ATP, there was no reserpine-sensitive dopamine uptake, whereas uptake was clearly reserpine sensitive at 0.1 mM and higher ATP concentrations, and the uptake stimulatory effect of ATP appeared saturated at a concentration of 2 mM (Fig. 2a–d). A two-way repeated measures ANOVA for the factors region and ATP revealed significantly higher rates of vesicular dopamine uptake in putamen than in caudate at saturating concentrations of ATP in controls (0.4 mM ATP, *t* = 2.216, *p* = 0.046; 2 mM ATP, *t* = 4.561, *p* < 0.001; Fig. 2e), but not in ILBD where this subregional difference was lost (Fig. 2f).

## Discussion

Idiopathic PD is a neurodegenerative disorder where progression spans a period of years and has presumably an asymptomatic preclinical phase before. The assumption of an asymptomatic preclinical phase is already based on early postmortem studies which, quite early after the fundamental discovery of the dopamine loss in PD^26^, suggest that striatal dopamine changes remain clinically silent until the threshold value of 60–80% dopamine loss is reached^27,28^. Regression analysis of the decline of pigmented neurons in the substantia nigra in relation to the duration of symptoms results in an estimation of 30% total loss at the time of PD symptom onset^29,30^. Measurements of the rate of PD progression by assessing striatal [^18^F]dopa metabolism with PET and UPDRS scoring on two occasions in individual PD patients suggested an average preclinical period between 3 and 7 years^31^. Since Lewy bodies, besides the death of dopaminergic neurons in the substantia nigra, are considered the hallmark of PD, cases with ILBD might be within this preclinical period given their substantia nigra neuron density being intermediate between control and PD^2,32,33^. Our findings of striatal dopamine being significantly reduced in putamen but not in caudate of ILBD cases support the evidence that ILBD might be, in fact, the preclinical status of not only parkinsonism in general but idiopathic PD in particular, given the special susceptibility of putamen to loss of dopamine markers in idiopathic PD^7–12^. Given that a recent postmortem study has found pathological α-synuclein in the enteric nervous system rarely without concomitant deposits in the central nervous system in ILBD^34^, the topographically limited changes in our ILBD cases indicate the vulnerability of dopaminergic terminals as key elements in idiopathic PD supporting a top-down pathological process^35^. The about 50% reduction of dopamine in putamen seems to be compensated in a way that clinical parkinsonism was absent in ILBD. Dopamine turnover, as determined by the ratio of HVA/dopamine, was increased in the putamen; however, it did not reach significance due to high variation with very high ratios in the two cases of ILBD with the lowest dopamine levels in the putamen. Loss of dopamine due to loss of dopaminergic nerve endings below 50% might be compensated without augmented dopamine synthesis in remaining neurons by concomitant loss of dopamine transporters potentiating volume transmission following the concept of “passive stabilization”^36^.

Other subregional characteristics in idiopathic PD striatum are a higher loss of dopaminergic innervation in the posterior than anterior putamen and asymmetry of dopaminergic innervation between hemispheres. Corresponding dopamine tissue levels and vesicular transport in ILBD would be highly interesting. However, routine dissection protocols are performed in a way that does not allow such analysis.

In a recent study on vesicular preparations from postmortem striatal tissue of cases with idiopathic PD, we reported a significantly reduced efficiency of vesicular dopamine transport, in particular in the putamen with an uptake rate per transport site reduced by 90% in dopamine nerve terminals. Similarly to this study, transport efficiency was determined by estimating both dopamine transport initial rate and steady-state binding of the specific label [^3^H]DTBZ to the VMAT2 sites in the preparation of synaptic vesicles of each control and idiopathic PD case and calculating the individual uptake rate per transport site. The observed dopamine transport defect appeared to be a specific feature of PD since it was found absent in tissue from *cynomolgus* monkeys with a comparable degree of degeneration of nigrostriatal dopaminergic terminals produced by MPTP^22^.

This defect of vesicular transport in PD can be taken as indicating increased cytosolic dopamine levels in PD striatum in vivo, which in turn would lower the threshold for several pathways of striatal dopaminergic neurodegeneration due to the production of dopamine-derived, cytotoxic reactive species triggering the “toxic-dopamine-cascade”^37–39^. On the other hand, the potential damage of high cytosolic dopamine in substantia nigra compacta compared to reticulata and ventrotegmental area is rescued by the synthesis of neuromelanin^40,41^. One could reason that similar findings in preclinical cases such as ILBD might indicate them as causative events in the PD disease process. However, in this study, dopamine uptake rates, [^3^H]DTBZ binding density, and turnover in vesicular preparations from caudate and putamen were not different in ILBD cases from controls, controls which nonetheless were in the same range of controls as in our previous study on PD. The number of control and ILBD cases studied was low; however, there was not even a tendency for reduction of mean values of uptake, binding sites, or turnover in ILBD as compared to control (Table 1), which does not suggest a different outcome with a higher sample size. As the vesicular transport dysfunction in our previous study was from patients with more than eight years of disease duration^22^, the vesicular transport dysfunction could have been due to secondary change by some more fundamental disturbance.

Since active transport into storage vesicles is driven by a transmembrane pH and electrochemical gradient generated by the vesicular H^+^-ATPase^42^, also called V-ATPase^43^, we reasoned that ATP-dependence of dopamine uptake might give additional insight and performed assays of putaminal and caudate preparations of synaptic vesicles of individual cases in parallel. The concentrations of ATP to stimulate dopamine uptake with saturation at 2 mM were in agreement with a study where ATP-dependence of another ATPase was reported, specifically ATPase activity in vascular membranes^44^. There was no difference in potency or maximal velocity of uptake between controls and ILBD; however, total dopamine uptake was significantly higher in putamen than in caudate at saturating concentrations of ATP in controls, a difference lost in ILBD. The higher vesicular dopamine uptake in putamen than caudate in head-to-head experiments might explain the higher dopamine tissue level in putamen than caudate in controls (*p* < 0.015 by paired Student’s *t*-test) since dopamine tissue levels essentially stem from dopamine within synaptic vesicles where dopamine depends on continuous accumulation by the vesicular monoamine transporter. The loss of the higher activity of the vesicular monoamine transporter in putamen than in caudate in ILBD might be an early event in the development of neurodegeneration in idiopathic PD. Since transport was determined by an accumulation assay, differences in energy-dependent storage might, in principle, contribute to the measured pmol/mg_prot_/min, although the short incubation time of 5 min, aiming at initial rates, makes this contribution less likely. Storage may, however, be a factor in dopamine tissue levels. Findings on reduced NADH-ubiquinone reductase and NADH cytochrome c reductase activities in the substantia nigra^45^ and down-regulated genes of subunits of the respiratory chain complex I in nigral dopamine neurons^46,47^ suggest disrupted energy metabolism in dopamine neurons in PD which might have a higher impact on dopamine in putamen with its higher levels and therefore higher energy-dependence. However, another very recent laser capture microdissection study on neuromelanin-positive cells revealed an upregulation of oxidative phosphorylation pathways in ILBD^48^, suggesting that reduced vesicular uptake and storage in putamen might be more related to changes in vesicular monoamine transporter 2 or vesicular H^+^-ATPase.

In conclusion, although our findings cannot rule out that ILBD is only an associated factor of PD, they give new support to the assumption that ILBD might be a preclinical stage of idiopathic PD. Obviously, this stage is only available for examinations postmortem since it is based on a neuropathological analysis. However, quite a few novel hypotheses about the neurodegenerative process in PD have been tested in postmortem tissue from PD patients, such as the increased expression of the vesicular glutamate transporter^49^, activation of NADPH oxidase 2^50^, or LRRK2 kinase^51^ in tyrosine hydroxylase positive neurons. ILBD postmortem tissue could be a valuable source for testing these hypotheses in idiopathic PD at an early stage with more validity for a causal role in the progression of the disease.

## Methods

### Human brain tissue

Tissue for this study was provided by the Newcastle Brain Tissue Resource, with full informed consent of donors. Brain tissue was obtained at autopsy and stored within the Newcastle Brain Tissue Resource (NBTR), with the full written informed consent of the donors, in accordance with the Newcastle University Ethics Board (The Joint Ethics Committee of Newcastle and North Tyneside Health Authority, reference: 08/H0906/136). Samples from the midcaudate (head) and midputamen (Supplementary Fig. 1) from six control subjects (52–88 years; two females, four males) and five ILBD subjects (70–96 years; three females, two males) without evidence in their records of any neurological or psychiatric disorder were stored at −80 °C until analysis. The standardized neuropathological assessment was performed for all cases and included the National Institute on Aging-Alzheimer’s Association (NIA-AA) criteria^52^ (inclusive of Thal phases of Aβ deposition, Braak staging of neurofibrillary tangle (NFT) pathology and Consortium to Establish a Registry for Alzheimer’s Disease (CERAD) scoring of the density of neuritic plaques) and McKeith Lewy body stage^53^, LBD-related neuropathological findings of the subjects, diagnosed according to the Lewy Pathology Consensus Criteria^54^ are shown in Table 2. The mean age (±SEM) of the control and ILBD group (75 ± 6 years, range 52–88 years and 82 ± 5, range 70–96 years, respectively) did not differ significantly, nor did the postmortem delay (=death to autopsy interval; controls: 51 ± 15 h, range: 25–102 h; ILBD: 54 ± 15 h range: 16–98 h).

**Table 2:** General characteristics of control and ILBD cases.

Disease code	Age at death/sex	Postmortem delay (h)	Neuropathological diagnosis (semiquantitative Lewy body pathology score)
Co	88/M	26	No LP
Co	64/M	93	No LP
Co	88/M	28	No LP
Co	80/F	25	No LP
Co	80/F	31	No LP
Co	52/M	102	No LP
ILBD	74/F	53	Brainstem predominant LP (dmX: 1, LC: 1)
ILBD	70/M	72	Brainstem predominant LP (dmX: 2, LC: 1)
ILBD	96/F	29	Limbic LP (dmX: 1, LC: 2, SN: 2, hippocampus: 1)
ILBD	80/M	16	Brainstem predominant LP (dmX: 1, LC: 2, SN: 1)
ILBD	89/F	98	Brainstem predominant LP (dmX: 1, LC: 2, SN: 1)

Neuropathological diagnosis according to the Lewy Pathology Consensus Criteria^54^. Semiquantitative scores are only provided for areas with Lewy pathology; other areas were assessed but did not show any Lewy pathology. Neuronal loss in the substantia nigra was mild in all cases (<30%).

*Co* control, *ILBD* incidental Lewy body disease, *LP* Lewy pathology, *dmX* dorsal motor nucleus of nervus vagus, *LC* locus ceruleus, *SN* substantia nigra.

### Determination of monoamine neurotransmitters and dopamine metabolites

Distinct samples of 20–50 mg from caudate and putamen frozen and kept at −80 °C were ultrasonicated from all subjects with an ultrasonic probe sonicator in 25 volumes of ice-cold perchloric acid, sodium bisulfite, and 3,4-dihydroxybenzylamine as internal standard (final concentration 0.1 M, 0.4 mM, and 25 µg/l, respectively). For determination of noradrenaline and dopamine, homogenates were centrifuged at 16,100 × *g* for 10 min at 4 °C, and 50 µl of supernatant was extracted with alumina oxide (10 g/l) in 1 M Tris-HCl, pH 8.6, and after washing with H_2_O and desorption with 0.2 ml 0.1 M perchloric acid containing 0.4 mM sodium bisulfite; 100 µl was injected into a high-performance liquid chromatography system with electrochemical detection (HPLC/ED) with a sodium phosphate mobile phase with l-octane sulfonic acid as described previously^25,55^. For determination of serotonin, 3,4-dihydroxyphenylacetic acid, and homovanillic acid, the supernatant was diluted 1:4 with 0.1 M perchloric acid containing 0.4 mM sodium bisulfite, and 100 µl was injected directly into an HPLC/ED system with a sodium phosphate mobile phase with l-heptane sulfonic acid as described previously^25,55^.

### Preparation of synaptic vesicles

For performing vesicular uptake and [^3^HDTBZ]-binding from the same preparation, about 140–180 mg of striatal tissue were homogenized in 8 volumes of ice-cold 0.3 M sucrose containing 25 mM Tris (pH 7.4) and 10 µM pargyline, using seven up-down strokes in a glass Teflon Potter-type homogenizer and the whole further procedure was performed at 4 °C. The sucrose-homogenate was centrifuged at 1000 × *g* for 15 min, and the supernatant was recentrifuged at 20,000 × *g* for 30 min. The resulting pellet (“P2”) was subjected to osmotic shock by resuspension in 1 ml H_2_O, frozen for about 15 min, and, after thawing, homogenized in a total of 2 ml H_2_O. The aqueous samples were centrifuged at 22,000 × *g* for 15 min, and the osmolarity of the supernatant was readjusted by the addition of 1.3 M potassium phosphate buffer (pH 7.4) in 1/10 the volume. The supernatant of the 20,000 × *g* centrifugation (see above) was centrifuged at 100,000 × *g* for 30 min, and the resulting pellet was resuspended in the about 2.17 ml of the 22,000 × *g* supernatant readjusted to 0.13 M potassium phosphate, thus combining vesicles in the supernatant of the P2 pellet and in the hypoosmotically shocked P2 pellet. After incubation in a total volume of 3 ml assay buffer KP (0.13 M potassium phosphate, pH 7.4) in the presence of 2 mM MgATP in a 30 °C water bath for 4 min and centrifugation at 4 °C at 100,000 × *g* for 45 min the resulting pellet was resuspended in 4.8 ml KP. On each preparation, 1.5 ml were centrifuged at 4 °C at 153,000 × *g* for 45 min, and the pellet was resuspended in 100 µl of binding buffer SP (25 mM sodium phosphate, pH 7.7) and frozen at −80 °C until the [^3^H]DTBZ binding experiment and the rest of the preparation was used for vesicular uptake. Protein content was determined according to the Bradford method by a Bio-Rad assay kit.

For comparison of ATP-dependence of vesicular uptake in putamen vs. caudate, about 200–350 mg striatal tissues were homogenized, volumina were up-scaled accordingly, and the preparation was simplified in a way that the combined vesicles in the supernatant of the P2 pellet and in the hypoosmotically shocked P2 pellet were directly used for uptake experiments.

### Vesicular uptake

Uptake was performed in a total volume of 1.5 ml KP containing 0.1 µM [^3^H]dopamine (New England Nuclear GmbH, Vienna), various concentrations of MgATP (2 mM in the uptake experiments with parallel [^3^HDTBZ]-binding experiments) and 1 µM reserpine for determination of unspecific uptake as described previously^22^. Transport was initiated by placing the tubes in a 30 °C water bath and adding 0.5 ml vesicle suspension (obtained from about 15–20 mg striatal tissue) for 5 min. Uptake was terminated by the addition of 2.5 ml ice-cold KP and immediate filtration under vacuum onto Whatman GF/B filter paper pre-soaked in 1% polyethylenimine using a Brandel harvester. The filters were washed twice with an additional 3 ml of cold KP and, after shaking in a liquid scintillation cocktail at 55 °C for 1 h, analyzed for tritium radioactivity in a liquid scintillation counter.

### [^3^H]DTBZ binding

Binding of (±)-a-dihydrotetrabenazine [2-3H] (American Radiolabeled Chemicals, Inc., Hartmann Analytic GmbH, Braunschweig, Germany) was performed on 25 µl thawed preparation in SP at a total volume of 25 µl for 90 min at 30 °C as described previously^22^.

### Data and statistical analysis

Data are reported as mean ± SE. Statistical analysis was done for neurotransmitter tissue levels using two-way analysis of variance for the factors disease (ILBD or control) and region (caudate or putamen) and for comparison of uptake in the putamen and caudate in the various control and ILBD cases using two-way repeated measures ANOVA (two factor repetition) for the factors region (caudate or putamen) and ATP (0, 0.1, 0.4, or 2 mM), both tests followed by pairwise multiple comparison procedures (Holm–Sidak method) after data passed the normality test (Shapiro–Wilk) and the equal variance test (Brown–Forsythe).

### Reporting summary

Further information on research design is available in the Nature Research Reporting Summary linked to this article.

## Supplementary information

Supplementary Figure 1

Reporting Summary

## Data Availability

The data that support the findings of this study are available from the corresponding author.
