# Supplementary material for: Dopamine and vesicular monoamine transport loss supports incidental Lewy body disease as preclinical idiopathic Parkinson
Source: NPJ Parkinsons Dis. 2023 Jun 15;9:89. doi: 10.1038/s41531-023-00514-z (PMC10272141; doi:10.1038/s41531-023-00514-z)
Supplement: Supplementary file 1 — Supplementary Figure 1 [file 41531_2023_514_MOESM1_ESM.docx]

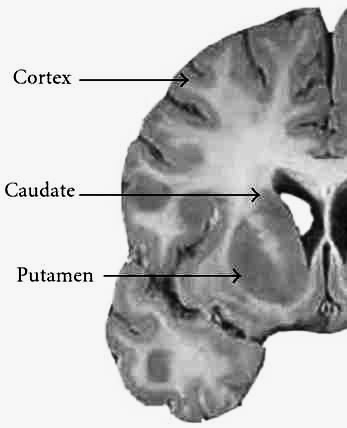


Supplementary Figure 1 **Midcaudate *vs.* midputamen.** Left half of a coronal slice of a human brain cut rostrally from globus pallidus.
